# Understanding the mental health and intention to leave of the public health workforce in Canada during the COVID-19 pandemic: A cross-sectional study

**DOI:** 10.1186/s12889-024-19783-1

**Published:** 2024-08-29

**Authors:** Emily Belita, Sarah E. Neil-Sztramko, Vanessa De Rubeis, Sheila Boamah, Jason Cabaj, Susan M. Jack, Cory Neudorf, Clemence Ongolo Zogo, Carolyn Seale, Gaynor Watson-Creed, Maureen Dobbins

**Affiliations:** 1 School of Nursing, McMaster University, 1280 Main Street West Hamilton, Hamilton, ON L8S 4K1 Canada; 2 Department of Health Research, Methods, Evidence, and Impact, McMaster University, 1280 Main Street West, Hamilton, ON L8S 4L8 Canada; 3 Department of Psychiatry & Behavioural Neurosciences, McMaster University, 1280 Main Street West, Hamilton, ON L8S 4L8 Canada; 4 Department of Community Health Sciences, University of Calgary, 3280 Hospital Drive NW, Calgary, AB T2N 4Z6 Canada; 5 College of Medicine, University of Saskatchewan, 107 Wiggins Road, Saskatoon, SK S7N 5E5 Canada; 6 Temerty Faculty of Medicine, University of Toronto, 1 King’s College Circle, Toronto, ON M5S 1A8 Canada; 7 Faculty of Medicine, Dalhousie University, 5849 University Ave, Halifax, NS B3H 4R2 Canada; 8 National Collaborating Centre for Methods and Tools, McMaster Innovation Park, 175 Longwood Rd. S., Suite 210a, Hamilton, ON L8P 0A1 Canada

**Keywords:** Public health workforce, COVID-19 pandemic, Mental health, Intention to leave

## Abstract

**Background:**

There is limited evidence about the mental health and intention to leave of the public health workforce in Canada during the COVID-19 pandemic. The objectives of this study were to determine the prevalence of burnout, symptoms of anxiety and depression, and intention to leave among the Canadian public health workforce, and associations with individual and workplace factors.

**Methods:**

A cross-sectional study was conducted using data collected by a Canada-wide survey from November 2022 to January 2023, where participants reported sociodemographic and workplace factors. Mental health outcomes were measured using validated tools including the Oldenburg Burnout Inventory, the 7-item Generalized Anxiety Disorder scale, and the 2-item Patient Health Questionnaire to measure symptoms of depression. Participants were asked to report if they intended to leave their position in public health. Logistic regression was used to estimate adjusted odds ratios (aOR) and 95% confidence intervals (95% CI) for the associations between explanatory variables such as sociodemographic, workplace factors, and outcomes of mental health, and intention to leave public health.

**Results:**

Among the 671 participants, the prevalence of burnout, and symptoms of depression and anxiety in the two weeks prior were 64%, 26%, and 22% respectively. 33% of participants reported they were intending to leave their public health position in the coming year. Across all outcomes, sociodemographic factors were largely not associated with mental health and intention to leave. However, an exception to this was that those with 16–20 years of work experience had higher odds of burnout (aOR = 2.16; 95% CI = 1.12–4.18) compared to those with ≤ 5 years of work experience. Many workplace factors were associated with mental health outcomes and intention to leave public health. Those who felt bullied, threatened, or harassed because of work had increased odds of depressive symptoms (aOR = 1.85; 95% CI = 1.28–2.68), burnout (aOR = 1.61; 95% CI = 1.16–2.23), and intention to leave (aOR = 1.64; 95% CI = 1.13–2.37).

**Conclusions:**

During the COVID-19 pandemic, some of the public health workforce experienced negative impacts on their mental health. 33% of the sample indicated an intention to leave their role, which has the potential to exacerbate pre-existing challenges in workforce retention. Study findings create an impetus for policy and practice changes to mitigate risks to mental health and attrition to create safe and healthy working environments for public health workers during public health crises.

**Supplementary Information:**

The online version contains supplementary material available at 10.1186/s12889-024-19783-1.

## Background

The public health workforce played a critical role as the first line of defense in the COVID-19 pandemic response [1, 2]. Public health workers assumed a multitude of essential roles and functions that included but was not limited to surveillance, case management, contact tracing, immunizations, risk communication, policy and protocol development, and health education [2–4]. These roles were performed within an already strained public health system that has been marred by historical divestments in public health staffing and resources [5, 6].

Despite the critical role of public health, much of the existing evidence on work experiences throughout the COVID-19 pandemic has focused on health care workers in acute care settings. Of the limited and emerging research, it has been revealed that some public health workers’ daily responsibilities were executed in difficult work environments fraught with heavy workloads, long working hours, constant redeployment and changing roles, limited resources, and insufficient staff to complete assigned work [7, 8]. Additionally, public health leaders and workers were spotlighted throughout the pandemic as they were responsible for communicating and enforcing the institution of public health measures with the public [2, 9]. In the United States (US), public health workers have reported experiences of criticism and backlash, as well as reports of bullying and harassment [1, 2, 9, 10]. Similarly in a study of public health workers in Canada, incidents of harassment including yelling, name calling, and rudeness, attributed to public health mandates and vaccine access, have been reported [11].

Several systematic reviews have focused on the impact of working during the COVID-19 pandemic on the mental health of health care professionals in hospitals or general practice settings, reporting a high prevalence of stress, anxiety, depression, insomnia, and burnout [12–14]. There have been limited studies exploring the impact of challenging work circumstances in a public health and pandemic context. Of studies that explored the prevalence of mental health outcomes among public health workers, most were conducted in the US with a few emerging from other countries. Across US studies conducted during the COVID-19 pandemic, the prevalence of adverse mental health outcomes ranged from 30.3 to 41% for anxiety and 29.1–30.8% for depression among public health workers [15–18]. Capturing a longitudinal perspective on these trends, one US study using a subsample of 85 public health workers reported decreased but persistent rates of anxiety (46.3% vs. 23.2%) and depression (37.8% vs. 26.8%) between 2020 and 2021 [19]. As well, two studies from China reported similar rates of anxiety (20.6-49.2%) and depression (27.1-45.7%) among frontline public health workers [20, 21]. One study from Canada (*n* = 2,055), conducted during a later pandemic stage (November 2022 to January 2023), reported a burnout prevalence of 79% among local, provincial, and national public health workers [11].

Notably, poor mental health and burnout have been associated with higher intent to leave among health care workers [22, 23]. Only one Canadian study thus far, has explored the association between intention to leave and burnout among public health workers during the COVID-19 pandemic, reporting an increased odds of intending to leave or retire early for those experiencing burnout (aOR = 6.13; 95% CI = 3.71–10.13) [11]. As intent to leave can be a potential precursor for actual workplace departure, it is of critical concern within an already strained public health system [24]. Response to the COVID-19 pandemic has impacted the career trajectory of US public health workers, with a larger proportion reporting intention to leave within one to two years versus pre-pandemic numbers [17]. Signs of intention to leave, which serves as a potential precursor for actual workplace departure, deserves critical attention given the downstream implications to available public health workforce capacity in the future. The loss of a substantial proportion of a skilled and experienced workforce weakens the infrastructure of the public health system leaving major gaps in its ability to respond to emerging public health crises and ongoing population health issues [2].

To date, there has not been a study in Canada to investigate mental health outcomes including symptoms of anxiety and depression, burnout, and intention to leave, among the public health workforce in the context of the COVID-19 pandemic and associations with various socio-demographic and workplace factors. Given that a resilient public health system is contingent on a competent, stable, and healthy workforce, attention toward the current state of the public health workforce in Canada is warranted to support funding and organizational human resource decision-making. To this end, the aims of this study were to determine the prevalence of burnout, symptoms of anxiety and depression, and intention to leave public health among the Canadian public health workforce, and to determine associations with individual characteristics and workplace factors.

## Methods

### Design, setting, and sample

A cross-sectional study was conducted using data collected from an online anonymous survey administered in English and French from November 2022-January 2023. Public health professionals with advanced public health or discipline-specific education/training (e.g., nurses, epidemiologists) or other workers (e.g., family home visitors), who were employed in local public health units or regional health authorities in Canada prior to March 2020 and for ≥ 8 months during the COVID-19 pandemic were eligible to participate in this study. Those who were employed prior to the pandemic were prioritized within this sample given that during the COVID-19 pandemic many public health workers were hired on short term contracts which would have substantial implications on intention to leave. Given this, it was important to prioritize trends among the established public health workforce that had roles prior to the pandemic and who would play integral roles in pandemic recovery once contracts ended. Participants were recruited through online communication channels (e.g., social media, email) of national and provincial public health organizations across Canada.

### Measures

#### Sociodemographic and workplace characteristics

Participants were asked to report their age, self-identified gender (woman; man; non-binary; if your gender is not listed, please describe; prefer not to answer), ethnicity (Asian; Black/African; Hispanic; Indigenous; Mixed, White/Caucasian; other; prefer not to answer), and education level. Participants were also asked to report their current role/occupation, field of practice within public health, position level, work location in Canada, and years worked.

#### Workplace stressors and resources

It is well accepted that employment circumstances can be influenced by both demands and resources [25]. Job demands are defined as a physical, social or organizational workplace stressor that necessitates increased and ongoing effort, while job resources are the supportive job aspects that help to reduce these demands and promote professional and personal development [25]. The interplay of these two concepts of demands and resources, can be associated with outcomes of disengagement or exhaustion (burnout) [25]. Two questions were used to evaluate workplace stressors and resources. The first question related to workplace stressors was: please indicate if while working in public health throughout the COVID-19 pandemic you ever experienced/felt any of the following (e.g., felt inadequately compensated for work; received job-related threats because of work). Response options were yes or no across nine developed items used in a previous US public health workforce study and treated as dichotomous data [15]. The second focused on the adequacy of workplace resources: thinking about your workplace environment overall while working throughout the COVID-19 pandemic, please rate your level of agreement with the following items (e.g., I always find new and interesting aspects in my work; there are days when I feel tired before I arrive at work). Eighteen items in this question originated from the US-based Public Health Workforce Interest and Needs Survey (PH WINS) [26]. Response options were on a 5-point Likert scale ranging from strongly disagree to strongly agree. In line with the original developers of this scale, a summed score was not used and each item was treated as an individual variable [26].

#### Mental health outcomes

Participants were asked to report burnout, and symptoms of anxiety and depression in the past two weeks and during the first wave of the pandemic. The Oldenburg Burnout Inventory (OLBI) was administered to measure participants’ job burnout [27]. The OLBI consists of 16 items divided into two 8-item subscales of exhaustion and disengagement. Items were rated on a 4-point scale ranging from 1-strongly agree to 4-strongly disagree, with negatively worded items being reverse-scored. For each subscale, items were summed, and an average score was calculated. A cut-off of ≥ 2.1 for disengagement and ≥ 2.25 for exhaustion was applied, based on scores above or below one standard deviation of the mean as used in previous literature [25, 28, 29]. Overall burnout was defined by the presence of both disengagement and exhaustion. The 7-item Generalized Anxiety Disorder (GAD-7) scale was administered to measure symptoms of anxiety, with higher scores indicating increased anxiety severity and where a score of ≥ 10 was considered to be indicative of potential clinical cases of GAD [30]. Items were rated on a 4-point scale from 0-not at all to 3-nearly every day. For depressive symptoms, the validated 2-item Patient Health Questionnaire (PHQ-2) was administered using a scale of 0-not at all to 3-nearly every day. A cut-off of ≥ 3 was used to indicate potential depression requiring further screening [31].

#### Intention to leave public health

A single-item adapted from a US national workforce survey assessed intention to leave public health to explore the potential future of the public health workforce. Participants were asked, “Are you considering leaving your position/organization in public health within the next year, and if so, why?” [26]. Response options were coded as: no (including not intending to leave job or intending to take a job in another public health unit or regional health authority) or, yes (including to take another job not in public health; to retire) and, unsure. Participants who reported they were unsure were marked as missing for the analysis. Participants were also asked if their intention to leave was influenced by their experiences working throughout the pandemic (no/yes).

### Statistical analysis

All analyses were conducted using SAS 9.4. Descriptive statistics were calculated for sociodemographic (age, gender, ethnicity, and education) and workplace characteristics (current role/occupation, field of practice, position level, work situated, and years worked), and workplace factors using frequency and percentage or mean and standard deviation.

To determine the associations between sociodemographic characteristics, workplace characteristics and workplace stressors with mental health in the past two weeks and intention to leave public health, we used univariate and multivariate logistic regression to estimate odds ratios (ORs) and 95% confidence intervals (CIs). To maintain a reasonable scope for this paper, associations with workplace stressors were reported and, in the future, associations with workplace resources will be explored. Due to small cell sizes, only some demographic/workplace variables were included in the multivariate regression models as explanatory variables. We ran unadjusted models (Supplementary Material) and models adjusted for age, gender, ethnicity, and education. Since there was minimal missing data across covariates, including explanatory and outcome variables, complete case analysis was used. These variables were identified a priori based on previous literature [15, 17, 32]. A secondary analysis was conducted where models were run to determine the associations between sociodemographic, workplace characteristics and stressors with mental health during the first wave of the pandemic.

## Results

Among the 671 public health workers in Canada who completed the survey, 7% were aged 18–29 years, 31% aged 30–38 years, 32% aged 40–49 years and the remaining 30% were 50 + years of age. Most participants self-identified their gender as woman (*n* = 588; 89%) and were of white ethnicity (*n* = 547; 84%). There was variation in the different roles/employment in which participants reported that they currently worked in, with the highest proportion reporting their current role as a nurse (*n* = 275; 41%). Similarly, there was variation in position level, with just over half reporting they were a front-line public health/community provider (*n* = 382; 57%). Most participants worked in Ontario (*n* = 516; 77%). A complete description of sociodemographic and workplace characteristics can be found in Table 1.

**Table 1:** Sociodemographic and workplace descriptive characteristics of study sample (*n* = 671)

Characteristics	*N* (%)
**Sociodemographic**	
Age	
18–29 years	46 (7%)
30–39 years	205 (31%)
40–49 years	213 (32%)
50 + years	201 (30%)
Prefer not to answer	6
Gender	
Woman	588 (89%)
Man	71 (11%)
Prefer not to answer/other	12
Ethnicity	
White	547 (84%)
Asian, Black/African, Hispanic, Indigenous, Mixed, Other)	103 (16%)
Prefer not to answer	21
Education	
Some college/no degree	26 (4%)
Bachelor’s degree	344 (51%)
Master’s degree	213 (32%)
Doctorate/professional degree	45 (7%)
Other	43 (6%)
Years worked	
0 to 5	144 (22%)
6 to 10	129 (19%)
11 to 15	124 (19%)
16 to 20	110 (16%)
21 or more	161 (24%)
Prefer not to answer	3
**Workplace descriptive characteristics**	
Current role/employment	
Administrator	35 (5%)
Physician/dentist	28 (4%)
Dietitian/nutritionist	18 (3%)
Epidemiologist/health analyst	14 (2%)
Family home visitor	10 (1%)
Health promotion specialist	46 (7%)
Nurse	275 (41%)
Policy advisor/policy analyst	20 (3%)
Program evaluator/planner	11 (2%)
Program manager/supervisor	87 (13%)
Public health educator	12 (2%)
Public health inspector	69 (10%)
Dental hygienist/assistant	10 (1%)
Administrative assistant	15 (2%)
Other	21 (3%)
Position level	
Administration assistant	28 (4%)
Medical officer of health (including chief and associate)	26 (4%)
Consultant	15 (2%)
Front-line public health/community provider	382 (57%)
Program/project management staff	130 (19%)
Senior management/administration (e.g., director, executive)	39 (6%)
Middle management	31 (5%)
Other	20 (3%)
Work situated	
Atlantic	25 (4%)
Quebec	12 (2%)
Ontario	516 (77%)
Prairies	80 (12%)
British Columbia	23 (3%)
Territories	14 (2%)
Prefer not to answer	1

### Prevalence of mental health and intention-to-leave public health

**Fig. 1:**
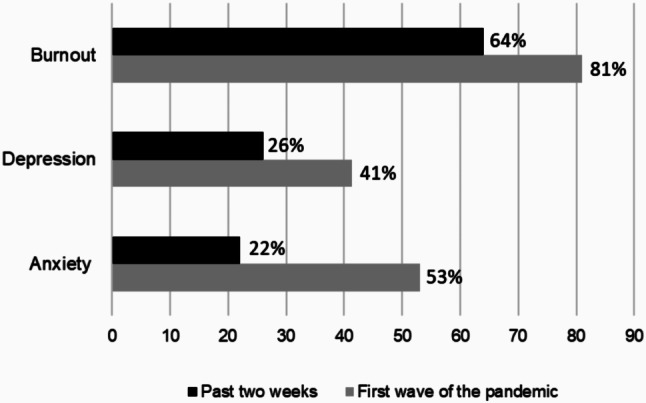
Prevalence of mental health symptoms during the first wave of the pandemic, and past two weeks among the Canadian public health workforce

During the past two weeks, the prevalence of burnout, depression and anxiety symptoms were 64%, 26%, and 22%, respectively. Prevalence of burnout, depression, and anxiety symptoms from the first wave of the pandemic (March 2020 to Fall 2020), was 81%, 41%, and 53%, respectively (Fig. 1).

**Fig. 2:**
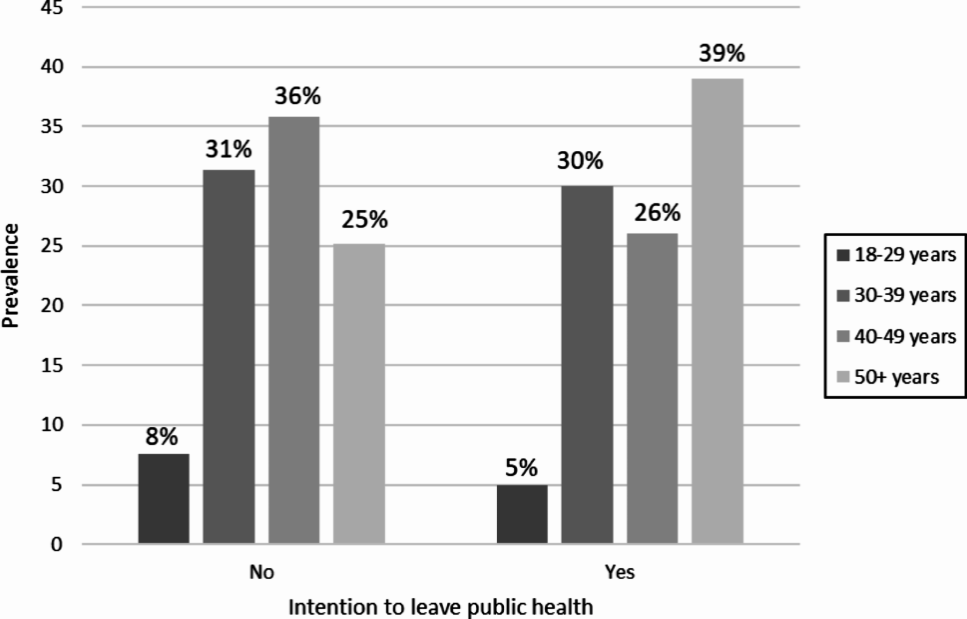
Prevalence of age among the Canadian public health workforce (*n* = 578), stratified by intention to leave public health

Approximately 33% of participants reported they had intentions to leave their public health position (*n* = 193), with the highest proportion among people who were aged 50+ (*n* = 74; 39%) (Fig. 2). Reasons for intending to leave public health include they intended to take a job outside of public health (*n* = 138) or to retire (*n* = 55). The majority of people also reported their intention to leave was influenced by experiences during the pandemic (*n* = 158; 82%).

### Associations between sociodemographic characteristics and mental health, and intention to leave public health

Socio-demographic factors were largely not associated with mental health or intention to leave. However, an exception to this was those participants with 16 to 20 years of work experience, who demonstrated increased odds of disengagement (aOR = 2.44; 95% CI = 1.07–5.58), exhaustion (aOR = 2.84; 95% CI = 1.40–5.76), and overall burnout (aOR = 2.16; 95% CI = 1.12–4.18) compared to those with 0–5 years of work experience. As well, those aged 50 + years experienced an increased odds of intention to leave (aOR = 2.35; 95% CI = 1.04–5.31). Those experiencing anxiety symptoms (aOR = 2.60; 95% CI = 1.69-4.00), depression symptoms (aOR = 2.06; 95% CI = 1.37–3.08), and burnout (aOR = 2.15; 95% CI = 1.45–3.17), had higher odds of intention to leave. Similar associations were observed among adjusted (Table 2) and unadjusted estimates, with the exception of a non-significant association with overall burnout in unadjusted estimates (Additional File 1).

**Table 2:** Adjusted^1^ association between sociodemographic characteristics and mental health in the past two weeks, and intention-to-leave public health

	Anxiety Symptoms Adjusted OR (95% CI)	Depression Symptoms Adjusted OR (95% CI)	Disengagement Adjusted OR (95% CI)	Exhaustion Adjusted OR (95% CI)	Burnout Adjusted OR (95% CI)	Intention-to-leave public health Adjusted OR (95% CI)
Age						
18–29 years	1.00	1.00	1.00	1.00	1.00	1.00
30–39 years	0.80 (0.37–1.73)	0.82 (0.39–1.72)	1.38 (0.66–2.86)	0.84 (0.40–1.74)	0.97 (0.49–1.92)	1.52 (0.67–3.44)
40–49 years	1.05 (0.49–2.26)	0.94 (0.45–1.98)	1.62 (0.77–2.40)	0.95 (0.45–1.98)	1.06 (0.53–2.10)	1.08 (0.47–2.47)
50 + years	0.73 (0.34–1.59)	1.17 (0.56–2.44)	1.35 (0.65–2.81)	0.74 (0.36–1.54)	0.89 (0.45–1.75)	**2.35 (1.04–5.31)**
Gender						
Man	1.00	1.00	1.00	1.00	1.00	1.00
Woman	1.20 (0.60–2.36)	0.84 (0.47–1.50)	1.68 (0.93–3.01)	1.49 (0.87–2.57)	1.62 (0.95–2.75)	1.05 (0.59–1.89)
Ethnicity						
White	1.00	1.00	1.00	1.00	1.00	1.00
Other	0.95 (0.56–1.61)	0.94 (0.57–1.55)	1.10 (0.65–1.86)	1.08 (0.67–1.73)	1.12 (0.71–1.76)	0.89 (0.54–1.45)
Education						
Some college/no degree	1.87 (0.97–3.60)	0.78 (0.39–1.56)	0.93 (0.46–1.88)	0.84 (0.40–1.74)	0.87 (0.48–1.60)	0.77 (0.37–1.59)
Bachelor’s degree	1.00	1.00	1.00	1.00	1.00	1.00
Master’s degree	1.28 (0.84–1.95)	1.07 (0.72–1.59)	0.79 (0.52–1.20)	0.95 (0.45–1.98)	0.82 (0.57–1.18)	1.28 (0.86–1.90)
Doctorate/professional degree	0.88 (0.36–2.12)	0.72 (0.32–1.61)	1.29 (0.55-3.00)	0.74 (0.36–1.54)	0.98 (0.49–1.95)	1.49 (0.73–3.03)
Years worked						
0 to 5	1.00	1.00	1.00	1.00	1.00	1.00
6 to 10	0.67 (0.36–1.27)	0.63 (0.34–1.17)	0.98 (0.54–1.79)	1.48 (0.85–2.58)	1.19 (0.70–2.02)	0.73 (0.409–1.33)
11 to 15	0.96 (0.50–1.83)	1.11 (0.60–2.05)	1.36 (0.70–2.65)	1.14 (0.64–2.02)	1.13 (0.64–1.98)	0.77 (0.41–1.46)
16 to 20	0.88 (0.43–1.83)	0.74 (0.37–1.47)	**2.44 (1.07–5.58)**	**2.84 (1.40–5.76)**	**2.16 (1.12–4.18)**	0.72 (0.35–1.47)
21 or more	0.92 (0.45–1.91)	0.71 (0.36–1.41)	0.91 (0.44–1.85)	1.29 (0.68–2.46)	1.17 (0.63–2.18)	1.06 (0.53–2.14)
Anxiety Symptoms						
Yes vs. No	–	-	–	–	-	**2.60 (1.69-4.00)**
Depression Symptoms						
Yes vs. No	-	–	–	–	-	**2.06 (1.37–3.08)**
Burnout						
Yes vs. No	-	-	–	–	–	**2.15 (1.45–3.17)**

^1^ Adjusted for age, gender, ethnicity, education

*Note* Bolded text indicates statistically significant findings

As well, in the first wave of the pandemic, women had increased odds of anxiety (aOR = 2.38; 95% CI = 1.36–4.16) and exhaustion (aOR = 2.13; 95% CI = 1.08–4.21) compared to men. Although findings within the last two weeks pertaining to anxiety (aOR = 1.20; 95% CI = 0.60–2.36), depression symptoms (aOR = 0.84; 95% CI = 0.47–1.50), burnout (aOR = 1.62; 95% CI = 0.95–2.75), disengagement (aOR = 1.68; 95% CI = 0.93–3.01), exhaustion (aOR = 1.49; 95% CI = 0.87–2.57), and intention to leave public health (aOR = 1.05; 95% CI = 0.59–1.89), compared to men were not statistically significant. Additional associations between sociodemographic characteristics, and mental health during the first wave of the pandemic are identified in Additional File 2.

### Prevalence of workplace resources related to the COVID-19 pandemic

Participants were asked about workplace resources related to the pandemic (Table 3). Many participants reported that they agreed/strongly agreed that they had an understanding about how their work relates to the organization’s goals and priorities (86%) and believed that the work they do is important (91%). Many participants also reported positive relationships with leadership reporting that they agreed/strongly agreed that they have a good working relationship with their supervisor (75%) and that their supervisor treats them with respect (77%). Notably, many participants disagreed/strongly disagreed that their training needs were assessed (47%), that they were provided sufficient training to utilize technology needed for their work (41%), and that their creativity and innovation was rewarded (45%).

**Table 3:** Prevalence of workplace resources related to the pandemic among the Canadian public health workforce

	Strongly disagree *n* (%)	Disagree *n* (%)	Neither agree or disagree *n* (%)	Agree *n* (%)	Strongly agree *n* (%)
I know how my work relates to the organization’s goals and priorities	18 (3%)	40 (6%)	40 (6%)	291 (44%)	280 (42%)
The work I do is important	13 (2%)	20 (3%)	23 (3%)	209 (31%)	405 (60%)
Creativity and innovation are rewarded	86 (13%)	213 (32%)	189 (28%)	141 (21%)	41 (6%)
Communication between senior leadership and employees is good in my organization	163 (24%)	198 (30%)	114 (17%)	146 (22%)	48 (7%)
Supervisors work well with employees of different backgrounds	37 (6%)	78 (12%)	165 (25%)	273 (41%)	114 (17%)
Supervisors in my work unit support employee development	52 (8%)	117 (18%)	126 (19%)	259 (39%)	113 (17%)
My training needs are assessed	84 (13%)	226 (34%)	140 (21%)	168 (25%)	50 (7%)
Employees have sufficient training to fully utilize technology needed for their work	76 (11%)	204 (30%)	127 (19%)	221 (33%)	41 (6%)
Employees learn from one another as they do their work	9 (1%)	25 (4%)	44 (7%)	349 (52%)	239 (36%)
My supervisor provides me with opportunities to demonstrate my leadership skills	53 (8%)	112 (17%)	121 (18%)	259 (39%)	122 (18%)
I have had opportunities to learn and grow in my position over the COVID-19 pandemic	73 (11%)	109 (16%)	91 (14%)	223 (33%)	172 (26%)
I feel completely involved in my work	31 (5%)	107 (16%)	135 (20%)	253 (38%)	142 (21%)
I am determined to give my best effort at work every day	21 (3%)	52 (8%)	98 (15%)	267 (40%)	226 (34%)
I am satisfied that I have the opportunities to apply my talents and expertise	72 (11%)	158 (24%)	110 (16%)	228 (34%)	102 (15%)
My supervisor and I have a good working relationship	24 (4%)	54 (8%)	93 (14%)	262 (40%)	229 (35%)
My supervisor treats me with respect	28 (4%)	40 (6%)	88 (13%)	268 (41%)	240 (36%)
I recommend my organization as a good place to work	64 (10%)	98 (15%)	159 (24%)	225 (34%)	121 (18%)
My organization prioritizes diversity, equity, and inclusion	35 (5%)	73 (11%)	162 (24%)	251 (38%)	141 (21%)

### Prevalence of workplace stressors during the COVID-19 pandemic and associations with mental health and intention to leave

Public health workers experienced a myriad of workplace stressors during the COVID-19 pandemic. Most stressors were prevalent, with the highest proportion of people reporting they felt overwhelmed by workload or family/work balance (*n* = 609; 91%). Most participants reported feeling inadequately compensated for work (*n* = 484; 73%) and unappreciated at work (*n* = 483; 72%). Some of the public health workers in our sample also received job-related threats (*n* = 235; 35%), experienced stigma/discrimination because of their work (*n* = 360; 54%), and felt bullied, threatened, or harassed because of their public health role (*n* = 360; 54%; Table 4).

Generally, many stressors were associated with anxiety and depression symptoms, burnout, disengagement, and exhaustion subscales, and intention to leave public health for adjusted (Table 4) and unadjusted estimates (Additional File 3). For instance, those who reported they felt inadequately compensated for their work had higher odds of anxiety (aOR = 4.86; 95% CI = 2.64–8.97), depression symptoms (aOR = 3.40; 95% CI = 2.06–5.60), burnout (aOR = 2.64; 95% CI = 1.83–3.81) and intention to leave public health (aOR = 1.84; 95% CI = 1.20–2.82). Increased odds of anxiety (aOR = 2.20; 95% CI = 1.35–3.59), depression symptoms (aOR = 2.50; 95% CI = 1.57–3.97), burnout (aOR = 2.61; 95% CI = 1.82–3.76), and intention to leave (aOR = 2.49; 95% CI = 1.60-3.87), were also demonstrated among those who reported feeling unappreciated at work. As well, those who felt bullied, threatened, or harassed because of work had increased odds of depression symptoms (aOR = 1.85; 95% CI = 1.28–2.68), burnout (aOR = 1.61; 95% CI = 1.16–2.23), and intention to leave (aOR = 1.64; 95% CI = 1.13–2.37). Stressors also tended to be significantly associated with mental health outcomes during the first wave of the pandemic (Additional File 4).

**Table 4:** The prevalence of workplace stressors and the adjusted^1^ association^2^ with mental health in the past two weeks, and intention-to-leave public health

	Prevalence of reported stressor *n* (%)	Anxiety Symptoms Adjusted OR (95% CI)	Depression Symptoms Adjusted OR (95% CI)	Disengagement Adjusted OR (95% CI)	Exhaustion Adjusted OR (95% CI)	Burnout Adjusted OR (95% CI)	Intention-to-leave public health Adjusted OR (95% CI)
Felt overwhelmed by workload or family/work balance	609 (91%)	**2.36 (1.01–5.48)**	**3.43 (1.42–8.30)**	**3.17 (1.77–5.70)**	**3.25 (1.83–5.77)**	**3.00 (1.69–5.33)**	1.21 (0.63–2.30)
Felt disconnected from family and friends because of workload	530 (79%)	**2.66 (1.47–4.81)**	**2.72 (1.58–4.67)**	**2.15 (1.39–3.32)**	**2.76 (1.83–4.14)**	**2.83 (1.89–4.23)**	**1.80 (1.12–2.90)**
Felt inadequately compensated for work	484 (73%)	**4.86 (2.64–8.97)**	**3.40 (2.06–5.60)**	**2.13 (1.42–3.19)**	**2.25 (1.55–3.27)**	**2.64 (1.83–3.81)**	**1.84 (1.20–2.82)**
Felt unappreciated at work	483 (72%)	**2.20 (1.35–3.59)**	**2.50 (1.57–3.97)**	**2.71 (1.81–4.05)**	**2.30 (1.58–3.33)**	**2.61 (1.82–3.76)**	**2.49 (1.60–3.87)**
Experienced stigma or discrimination because of work	360 (54%)	**1.61 (1.09–2.40)**	**1.90 (1.30–2.77)**	**1.80 (1.22–2.65)**	**1.62 (1.14–2.29)**	**1.87 (1.34–2.61)**	**1.74 (1.20–2.53)**
Received job-related threats because of work	235 (35%)	1.36 (0.92–2.03)	**1.85 (1.28–2.68)**	**2.40 (1.53–3.76)**	**1.79 (1.23–2.62)**	**1.99 (1.39–2.86)**	**1.55 (1.06–2.26)**
Felt bullied, threatened, or harassed because of work	360 (54%)	1.38 (0.93–2.03)	**1.85 (1.28–2.68)**	**2.22 (1.51–3.27)**	1.36 (0.96–1.91)	**1.61 (1.16–2.23)**	**1.64 (1.13–2.37)**
Interacted often with the public	532 (80%)	0.79 (0.49–1.27)	1.12 (0.70–1.80)	0.96 (0.59–1.57)	1.12 (0.73–1.74)	1.12 (0.73–1.71)	1.45 (0.91–2.33)
Worried about workplace exposure to COVID-19	369 (55%)	1.14 (0.77–1.69)	1.24 (0.86–1.81)	1.31 (0.89–1.93)	**1.50 (1.06–2.13)**	**1.54 (1.10–2.15)**	1.25 (0.86–1.82)

^1^ Adjusted for age, gender, ethnicity, education

^2^ Reference group: Those who responded no to each workplace stressor

*Note* Bolded text indicates statistically significant findings

## Discussion

This study offers a beginning illustration of the state of mental health and intention to leave among a sample of public health workers in Canada during the COVID-19 pandemic. Findings reflect that some of the public health workforce experienced symptoms of anxiety and depression, burnout, and intention to leave in the context of challenging working conditions. The prevalence of adverse mental health outcomes in this study during the first wave of the pandemic for burnout, anxiety, and depression symptoms was 81%, 53%, and 41%, respectively. In the two weeks preceding survey completion, prevalence of burnout, anxiety symptoms, and depression symptoms was 64%, 22%, and 26%, respectively. In a study of 2,055 Canadian public health practitioners, burnout prevalence was comparatively higher (79%) [11] than our findings (64%) during a similar data collection period (November 2022 – January 2023). However, first wave prevalence rates of anxiety and depression symptoms in our study were higher compared to other studies in the US. Given we asked participants to retrospectively report these outcomes, recall bias likely influenced these rates. In a study of 354 public health workers from 35 states across the US, 39.6% and 29.4% reported symptoms of anxiety and depression, respectively, between August 2020 to January 2021 [16]. Similar findings were reported by Bryant-Genevier et al. [15, 18] in a large study of state, tribal, and territorial health department workers (*n* = 26,174) across the US in which half (53%) reported symptoms of at least one adverse mental health condition and 30.3% and 30.8% experienced anxiety and depression respectively between March and April 2021. Slightly lower rates of depression (27.1%) and anxiety (20.6%) were reported in a study of public health workers in China (*n* = 2,313), although this was during very early pandemic periods (February to March 2020) where the impact of working through the COVID-19 pandemic may not have yet been fully realized [20]. Variations in prevalence rates may be associated with the pandemic wave in which data was collected, differences in the implementation of public health measures and severity/volume of COVID-19 cases corresponding to different levels of response and workloads across geographic regions. Additionally, prevalence rates may also be impacted by different moderating factors at the individual (e.g., social, financial), workplace setting, and societal/cultural level that may influence risk for mental health challenges [16].

In our study, specific subgroups were identified as being at more risk of certain mental health outcomes, requiring more focused attention. For example, within the first wave of the pandemic, in our sample, women had higher odds of anxiety, and exhaustion compared to men, which are similar to results reported in Canada, the US and Japan; increased prevalence or odds of exhaustion [11] or burnout [17, 33] and increased risk of anxiety and depression among women public health workers compared to men have been noted [16]. Of note, in a study of Canadian public health workers, odds of exhaustion among women were significantly higher compared to men (aOR = 1.56; 95% CI = 1.09–2.25) [11]. Findings have importance for public health nurses, a largely female dominated field, who represent the largest professional group within the public health workforce [33]. Public health nurses experienced unique challenges during the COVID-19 pandemic given that their nursing designation was associated with work overload, expectations and organizational obligations to fulfil diverse and multiple types of work roles, and pressures to simultaneously staff the COVID-19 pandemic response efforts while maintaining mandated health promotion work [8].

As well, those with 16 to 20 years of work experience had twice the odds of disengagement, exhaustion, and overall burnout compared to those with less work experience in our study. This was similarly found in a cross-sectional study of Canadian public health workers in 2022/2023, in which those who had 10 to 19 years of work experience also had higher odds of burnout compared to those with less than 2 years experience (aOR = 2.45; 95% CI = 1.72–3.49) [11]. High burnout prevalence was also reported among a group of US public health workers (*n* = 225) with 15 + years of experience (63.5%) compared to those with less than 1 year of work experience (38.1%) [17]. In this same study, more experienced workers (10–14 years) were over four times as likely to report burnout versus those with less experience (< 1 year) [17]. Within these groups of higher work experience, it would be important to consider the potential that additional personal or household demands beyond the work environment may also play a role in influencing mental health outcomes.

Thirty-three percent of participants in our study identified that they were intending to leave their public health position/organization in the following year. This is slightly lower than findings reported in a study of US public health workers who were considering leaving their job because of the pandemic (44%) [34]. Since the COVID-19 pandemic, rates among public health professionals planning to leave or retire within the next two years has increased from 4.8 to 12% in the US [17]. Additional findings in our study identify that the highest proportion of those in the age group of ≥ 50 years were intending to leave and those aged 50 + years had almost twice the odds of intention to leave. These rates sound the alarm on attrition and workforce gaps of experienced public health workers across the US and Canada. Due to the temporary suspension of public health services and programs related to maternal-child, chronic disease, substance use, and other non-COVID infectious diseases (e.g., sexually transmitted infections, tuberculosis) during the COVID-19 pandemic [35, 36], a sufficient and expert public health workforce will be evermore critical in responding to imminent and growing population and community needs in a post-COVID era. At this juncture, recruitment and retention should be prioritized for funding bodies and health units to ensure a stable and competent public health workforce is available to deal with emerging public health crises.

Challenging work experiences and environments reported by public health workers in our study also mirror difficult conditions reported elsewhere. Our study found higher prevalence rates compared to a US study of 26,174 public health staff [15] related to overwhelming workloads (91% vs. 72%), receiving job-related threats (72% vs. 11.8%), and feeling bullied, threatened or harassed because of work (54% vs. 23.4%). These differences may be due to a relatively larger proportion of our study sample (80%) having frequent interactions with the public compared to under half (43%) of the US-based study sample [15] being in public facing roles. Differences may also be attributed to changes in perceptions of the public health workforce across different pandemic waves. Earlier in the pandemic workers were labelled as health care heroes, although as the pandemic progressed and more restrictive public health measures were implemented, hostility and mistrust from the public grew toward public health entities [9, 34, 37]. Growing concerns about workplace violence against public health workers have precipitated conversations and recommendations around their protection including the use of risk management training in health departments, conflict management and de-escalation strategies [34, 38].

Also concerning are the positive associations between workplace stressors plaguing most of our study sample and reported mental health outcomes and intention to leave. These relationships also held true among a sample of US public health workers in which experiencing any type or number of workplace violence event (e.g., bullying, harassment, job-related threats) was associated with increased prevalence risk for depression symptoms (PR = 1.95; 95% CI = 1.87–2.03), anxiety symptoms, (PR = 1.87; 95% CI = 1.80–1.94) and post-traumatic stress disorder (PR = 2.0; 95% CI = 1.93–2.07) [38]. A lack of appreciation at work was also associated with increased anxiety symptoms (aPR = 1.15; 95% CI = 1.10–1.20) and depression symptoms (aPR = 1.15; 95% CI = 1.10–1.21) in a study of US public health workers [39]. In comparison, our study found higher odds of anxiety (aOR = 2.20; 95% CI = 1.35–3.59) and depression (aOR = 2.50; 95% CI = 1.57–3.97) among those reporting feeling unappreciated at work.

Our study findings, in alignment with other public health workforce studies underscore alarming mental health needs and an anticipated exodus of experienced staff, precipitated by challenging working conditions throughout the COVID-19 pandemic. This calls for attention toward effective interventions to mitigate risks to the mental health of public health workers during pandemics and support the current workforce in pandemic recovery. The lack of significant associations between mental health outcomes and many of the socio-demographic variables but strong associations between mental health and workplace factors provides strong rationale for the need for organizational level interventions that foster positive mental health. An evidence synthesis of 28 reviews on strategies or interventions to support the mental health and resilience of frontline health care workers during the COVID-19 pandemic or previous pandemics determined that none of the reviews included studies in public health, although some of the strategies identified could potentially be modified for a public health setting [40]. Synthesis findings highlighted three main areas for consideration including: individual and team strategies centred on training and education, peer/social support and behaviour-based interventions targeting mental health; organization and management strategies addressing staffing and workloads, communication, positive workplace culture and effective leadership; and policies on pandemic preparedness, occupational health, and funding for mental health resources [40]. These strategies align well with a proposed framework by Preston et al. [41] to sustain resilience and promote the mental health of the public health workforce using a three-tiered system of intervening at the frontline staff level (Tier 1), the program and supervisor level (Tier 2), and at the senior management and executive levels (Tier 3). Preston et al. [41] further advocate for an upstream approach in the development of organizational policies and practices that set the tone for workplace wellness.

While this study provides one of the first explorations on various mental health outcomes and intention to leave among the public health workforce in Canada during the COVID-19 pandemic, there are limitations to consider. First, our study includes a small sample size limiting power to conduct certain analyses and may potentially explain some of the statistically non-significant findings and wide confidence intervals. However, the results are still important given the limited existing evidence on this topic and indications of concerning challenges experienced by the public health workforce that warrant further exploration. A second limitation considers the risk of sampling bias. It is important to note that it is possible that those who volunteered to participate in the survey may differ from individuals who decided not to participate based on socio-demographic characteristics or mental health and intention to leave outcomes of interest. Related to this, it is difficult to comment specifically on the representativeness of this sample given the lack of robust data on the public health workforce in Canada, which remains a longstanding challenge. A third sample limitation relates to the majority of participants being located in the province of Ontario. While this does represent the most populous province in Canada, there is still an under representation of perspectives and experiences from other provinces and territories and as such, the results may not be generalizable to the entirety of the public health workforce across Canada. In future public health workforce studies, more targeted recruitment approaches at the provincial level may help to remedy this particular challenge. Our team was also unable to conduct sub analyses based on race or ethnicity given the small size and homogeneity of our sample. A related limitation to this is that the response options provided to participants to capture their race did not align with updated Canadian guidance for collecting race-based data. We recognize this as an important area for future investigation to ensure this data is accurately collected and to also capture public health workers that self-identify as multiple races [15], as a higher prevalence of anxiety and depression has been noted among those that self-identify as multiple races in the US. In addition, given the small sample sizes across position and role categories, we were unable to conduct sub analyses pertaining to these factors, eliciting another future area of exploration comparing the influence of public health roles on mental health and intention to leave outcomes. We also were unable to explore characteristics associated with participants who reported they were unsure about their intention to leave public health. This may be an interesting area for future work, as this may be a distinct subgroup who share similar characteristics. Given that our sample inclusion criteria focused specifically on public health workers employed in local or regional public health organizations, we recognize that the results of this study may not generalize to those working in public health in the academic field or the federal government. As well, the use of a two-item measure to assess symptoms of depression may have limitations with respect to sensitivity or specificity compared to a longer measure such as the PHQ-9, however, for the purposes of this study, it was important to consider survey burden with this study population during data collection.

## Conclusion

Study findings provide a beginning perspective on the extent to which the public health workforce was impacted while working throughout the COVID-19 pandemic. Concerning and lingering rates of burnout, anxiety, and depression plaguing the public health workforce signal an important juncture for public health employers and funding bodies to strategically address these issues with impending population and community health needs on the horizon in post-COVID-19 pandemic recovery. With notions of older and experienced workers intending to leave their positions, the public health workforce is at compounded risk of instability. Urgent attention is needed to further understand workforce groups at most risk of mental health outcomes and attrition, and what specific interventions can mitigate these challenges in public health work environments. Our findings suggest that organizational factors and workplace experiences may play more of a critical role in influencing mental health outcomes and intention to leave among the public health workforce compared to individual characteristics. As such, it would be imperative for public health leaders to consider cultivating an environment for employees in which they feel valued, adequately compensated, and a sense of physical and emotional safety.

### Electronic supplementary material

Below is the link to the electronic supplementary material.

Supplementary Material 1: **Additional File 1**. Unadjusted association between sociodemographic characteristics, mental health in the past two weeks, and intention-to-leave public health

Supplementary Material 2: **Additional File 2**. The adjusted association between sociodemographic characteristics, mental health during the first wave of the pandemic

Supplementary Material 3: **Additional File 3**. The unadjusted association between workplace stressors and mental health in the past two weeks, and intention-to-leave public health

Supplementary Material 4: **Additional File 4**. The adjusted association between workplace stressors and mental health during the first wave of the pandemic

## Data Availability

The datasets supporting the conclusions of this article are available upon request from the corresponding author.

## Abbreviations

CI: Confidence Interval
GAD-7: 7-item Generalized Anxiety Disorder
OLBI: Oldenburg Burnout Inventory
OR: Odds ratio
aOR: Adjusted odds ratio
PHQ-2: 2-item Patient Health Questionnaire
PHQ-9: 9-item Patient Health Questionnaire
