# Supplementary material for: Understanding the mental health and intention to leave of the public health workforce in Canada during the COVID-19 pandemic: A cross-sectional study
Source: BMC Public Health. 2024 Aug 29;24:2347. doi: 10.1186/s12889-024-19783-1 (PMC11360311; doi:10.1186/s12889-024-19783-1)
Supplement: Supplementary file 1 — Supplementary Material 1: Additional File 1. Unadjusted association between sociodemographic characteristics, mental health in the past two weeks, and intention-to-leave public health [file 12889_2024_19783_MOESM1_ESM.docx]

**Additional File 1.** Unadjusted association between sociodemographic characteristics, mental health in the past two weeks, and intention-to-leave public health

|  | Anxiety Symptoms  Unadjusted OR (95% CI) | Depression Symptoms  Unadjusted OR (95% CI) | Disengagement  Unadjusted OR (95% CI) | Exhaustion  Unadjusted OR (95% CI) | Burnout  Unadjusted OR (95% CI) | Intention-to-leave public health  Unadjusted OR (95% CI) |
| --- | --- | --- | --- | --- | --- | --- |
| Age | | | | | | |
| 18-29 years | 1.00 | 1.00 | 1.00 | 1.00 | 1.00 | 1.00 |
| 30-39 years | 0.73 (0.35-1.53) | 0.75 (0.37-1.55) | 1.28 (0.62-2.63) | 0.79 (0.38-1.63) | 0.92 (0.47-1.79) | 1.40 (0.64-3.07) |
| 40-49 years | 0.92 (0.44-1.90) | 0.86 (0.42-1.76) | 1.60 (0.77-3.29) | 0.89 (0.43-1.84) | 1.04 (0.53-2.03) | 1.04 (0.47-2.28) |
| 50+ years | 0.68 (0.32-1.44) | 1.05 (0.52-2.14) | 1.37 (0.66-2.81) | 0.72 (0.35-1.49) | 0.88 (0.45-1.72) | **2.24 (1.03-4.48)** |
| Gender | | | | | | |
| Man | 1.00 | 1.00 | 1.00 | 1.00 | 1.00 | 1.00 |
| Woman | 1.14 (0.62-2.11) | 0.88 (0.51-1.52) | 1.55 (0.90-2.68) | 1.47 (0.88-2.44) | 1.53 (0.93-2.51) | 1.01 (0.59-1.73) |
| Ethnicity | | | | | | |
| White | 1.00 | 1.00 | 1.00 | 1.00 | 1.00 | 1.00 |
| Other | 0.98 (0.59-1.63) | 0.99 (0.61-1.61) | 1.12 (0.67-1.89) | 1.12 (0.71-1.79) | 1.17 (0.75-1.83) | 0.84 (0.52-1.35) |
| Education | | | | | | |
| Some college/no degree | 1.73 (0.91-3.29) | 0.78 (0.39-1.54) | 0.90 (0.45-1.80) | 0.90 (0.48-1.70) | 0.86 (0.47-1.55) | 0.82 (0.40-1.66) |
| Bachelor's degree | 1.00 | 1.00 | 1.00 | 1.00 | 1.00 | 1.00 |
| Master's degree | 1.33 (0.88-2.00) | 1.01 (0.69-1.48) | 0.75 (0.50-1.13) | 0.77 (0.53-1.12) | 0.80 (0.56-1.14) | 1.18 (0.80-1.72) |
| Doctorate or professional degree | 0.89 (0.40-2.00) | 0.68 (0.32-1.47) | 0.90 (0.43-1.90) | 0.63 (0.33-1.20) | 0.76 (0.40-1.43) | 1.30 (0.67-2.54) |
| Years worked | | | | | | |
| 0 to 5 | 1.00 | 1.00 | 1.00 | 1.00 | 1.00 | 1.00 |
| 6 to 10 | 0.62 (0.34-1.12) | 0.60 (0.34-1.07) | 0.92 (0.53-1.60) | 1.24 (0.74-2.08) | 1.03 (0.63-1.69) | 0.81 (0.46-1.42) |
| 11 to 15 | 0.96 (0.55-1.67) | 1.19 (0.70-2.03) | 1.33 (0.74-2.38) | 0.93 (0.56-1.56) | 1.00 (0.61-1.65) | 0.88 (0.50-1.54) |
| 16 to 20 | 0.88 (0.49-1.58) | 0.82 (0.46-1.45) | **3.40 (1.20-4.79)** | **1.94 (1.08-3.47)** | 1.68 (0.97-2.88) | 0.80 (0.45-1.43) |
| 21 or more | 0.74 (0.43-1.28) | 0.93 (0.56-1.55) | 0.93 (0.55-1.57) | 0.95 (0.59-1.53) | 0.95 (0.60-1.51) | 1.40 (0.85-2.32) |
| Anxiety Symptoms | | | | | | |
| Yes vs. No | – | **23.48 (14.71-37.47)** | – | – | **18.96 (8.22-43.70)** | **2.51 (1.67-3.78)** |
| Depression Symptoms | | | | | | |
| Yes vs. No | **23.48 (14.71-37.47)** | – | – | – | **20.97 (9.64-45.61)** | **2.30 (1.57-3.40)** |
| Burnout | | | | | | |
| Yes vs. No | **18.96 (8.22-43.70)** | **20.97 (9.64-45.61)** | – | – | – | **2.04 (1.40-2.97)** |

Note: Bolded text indicates statistically significant findings.
