# Supplementary material for: Understanding the mental health and intention to leave of the public health workforce in Canada during the COVID-19 pandemic: A cross-sectional study
Source: BMC Public Health. 2024 Aug 29;24:2347. doi: 10.1186/s12889-024-19783-1 (PMC11360311; doi:10.1186/s12889-024-19783-1)
Supplement: Supplementary file 2 — Supplementary Material 2: Additional File 2. The adjusted association between sociodemographic characteristics, mental health during the first wave of the pandemic [file 12889_2024_19783_MOESM2_ESM.docx]

**Additional File 2.** The adjusted^1^ association between sociodemographic characteristics, mental health during the first wave of the pandemic

|  | Anxiety Symptoms  Adjusted OR (95% CI) | Depression Symptoms  Adjusted OR (95% CI) | Disengagement  Adjusted OR (95% CI) | Exhaustion  Adjusted OR (95% CI) | Burnout  Adjusted OR (95% CI) |
| --- | --- | --- | --- | --- | --- |
| Age | | | | | |
| 18-29 years | 1.00 | 1.00 | 1.00 | 1.00 | 1.00 |
| 30-39 years | 0.43 (0.21-0.88) | 0.52 (0.27-1.01) | 0.90 (0.35-2.33) | 1.41 (0.52-3.78) | 1.37 (0.61-3.05) |
| 40-49 years | 0.58 (0.29-1.17) | 0.51 (0.26-0.98) | 1.50 (0.56-4.02) | 1.41 (0.52-3.78) | 1.67 (0.74-3.76) |
| 50+ years | 0.37 (0.18-0.75) | 0.41 (0.21-0.81) | 0.71 (0.28-1.83) | 0.98 (0.37-2.59) | 0.89 (0.41-1.96) |
| Gender | | | | | |
| Man | 1.00 | 1.00 | 1.00 | 1.00 | 1.00 |
| Woman | **2.38 (1.36-4.16)** | 1.35 (0.77-2.35) | 1.72 (0.89-3.30) | **2.13 (1.08-4.21)** | 1.76 (0.97-3.18) |
| Ethnicity | | | | | |
| White | 1.00 | 1.00 | 1.00 | 1.00 | 1.00 |
| Other | 0.75 (0.48-1.16) | 1.16 (0.74-1.81) | 0.87 (0.47-1.58) | 0.78 (0.41-1.48) | 0.78 (0.46-1.31) |
| Education | | | | | |
| Some college/no degree | 0.99 (0.55-1.80) | 1.09 (0.60-1.97) | 0.80 (0.35-1.84) | 0.61 (0.26-1.43) | 1.37 (0.61-3.05) |
| Bachelor's degree | 1.00 | 1.00 | 1.00 | 1.00 | 1.00 |
| Master's degree | 0.77 (0.54-1.09) | 0.67 (0.47-0.97) | 0.62 (0.38-1.03) | 0.78 (0.44-1.39) | 1.67 (0.74-3.76) |
| Doctorate/professional degree | 0.62 (0.31-1.25) | 0.67 (0.33-1.36) | 0.38 (0.17-0.85) | 0.44 (0.19-1.02) | 0.89 (0.41-1.96) |
| Years worked | | | | | |
| 0 to 5 | 1.00 | 1.00 | 1.00 | 1.00 | 1.00 |
| 6 to 10 | 1.32 (0.78-2.23) | 1.52 (0.88-2.62) | **2.39 (1.12-5.08)** | **3.55 (1.61-7.85)** | **1.99 (1.05-3.79)** |
| 11 to 15 | 1.41 (0.81-2.45) | 1.48 (0.82-2.67) | 1.75 (0.82-3.74) | **6.46 (2.59-16.15)** | **2.44 (1.21-4.92)** |
| 16 to 20 | 1.52 (0.82-2.83) | **2.03 (1.06-3.90)** | 1.68 (0.70-4.05) | **5.75 (2.28-14.52)** | **2.54 (1.17-5.52)** |
| 21 or more | 1.20 (0.65-2.22) | **2.26 (1.17-4.36)** | 1.61 (0.70-3.73) | **6.69 (2.78-16.11)** | **2.61 (1.25-5.47)** |
| Anxiety Symptoms | | | | | |
| Yes vs. No | – | - | – | – | **6.13 (3.75-10.03)** |
| Depression Symptoms | | | | | |
| Yes vs. No | - | – | – | – | **5.62 (3.24-9.74)** |
| Burnout | | | | | |
| Yes vs. No | - | - | – | – | – |

1. Adjusted for age, gender, ethnicity and education

Note: Adjustment for potential confounders slightly changed estimates, only adjusted results are presented given the minimal difference in estimates. Bolded text indicates statistically significant findings.
