# Supplementary material for: Understanding the mental health and intention to leave of the public health workforce in Canada during the COVID-19 pandemic: A cross-sectional study
Source: BMC Public Health. 2024 Aug 29;24:2347. doi: 10.1186/s12889-024-19783-1 (PMC11360311; doi:10.1186/s12889-024-19783-1)
Supplement: Supplementary file 3 — Supplementary Material 3: Additional File 3. The unadjusted association between workplace stressors and mental health in the past two weeks, and intention-to-leave public health [file 12889_2024_19783_MOESM3_ESM.docx]

**Additional File 3.** The unadjusted association between workplace stressors and mental health in the past two weeks, and intention-to-leave public health

|  | Anxiety Symptoms  Unadjusted OR (95% CI) | Depression Symptoms  Unadjusted OR  (95% CI) | Disengagement  Unadjusted OR (95% CI) | Exhaustion  Unadjusted OR (95% CI) | Burnout  Unadjusted OR (95% CI) | Intention-to-leave public health  Unadjusted OR (95% CI) |
| --- | --- | --- | --- | --- | --- | --- |
| Felt overwhelmed by workload or family/work balance | **2.28 (1.02-5.13)** | **3.49 (1.48-8.26)** | **2.73 (1.58-4.72)** | **3.06 (1.79-5.21)** | **2.84 (1.66-4.87)** | 1.13 (0.62-2.07) |
| Felt disconnected from family and friends because of workload | **2.47 (1.42-4.32)** | **2.78 (1.64-4.72)** | **2.16 (1.43-3.26)** | **2.71 (1.84-3.99)** | **2.78 (1.90-4.07)** | **1.67 (1.07-2.62)** |
| Felt inadequately compensated for work | **4.77 (2.62-8.68)** | **3.01 (1.87-4.86)** | **2.01 (1.36-2.97)** | **2.21 (1.54-3.17)** | **2.56 (1.80-3.64)** | **1.56 (1.04-2.33)** |
| Felt unappreciated at work | **1.99 (1.25-3.16)** | **2.27 (1.46-3.54)** | **2.62 (1.78-3.86)** | **2.27 (1.59-3.24)** | **2.64 (1.86-3.75)** | **2.30 (1.51-3.49)** |
| Experienced stigma or discrimination because of work | **1.54 (1.06-2.25)** | **1.80 (1.26-2.59)** | **1.79 (1.23-2.59)** | **1.69 (1.21-2.36)** | **1.87 (1.36-2.58)** | **1.59 (1.12-2.26)** |
| Received job-related threats because of work | 1.37 (0.94-2.01) | **1.96 (1.37-2.80)** | **2.39 (1.55-3.70)** | **1.80 (1.25-2.59)** | **1.99 (1.40-2.82)** | **1.56 (1.08-2.23)** |
| Felt bullied, threatened, or harassed because of work | 1.37 (0.95-2.00) | **1.90 (1.32-2.73)** | **2.15 (1.48-3.13)** | 1.36 (0.98-1.90) | **1.58 (1.15-2.17)** | **1.64 (1.16-2.34)** |
| Interacted often with the public | 0.77 (0.50-1.19) | 1.11 (0.72-1.72) | 1.03 (0.66-1.62) | 1.17 (0.78-1.75) | 1.17 (0.79-1.72) | 1.27 (0.82-1.95) |
| Worried about workplace exposure to COVID-19 | 1.16 (0.80-1.69) | 1.21 (0.85-1.72) | 1.40 (0.97-2.01) | **1.56 (1.12-2.17)** | **1.55 (1.12-2.13)** | 1.21 (0.85-1.72) |

Note: Bolded text indicates statistically significant findings.
