# Supplementary material for: Understanding the mental health and intention to leave of the public health workforce in Canada during the COVID-19 pandemic: A cross-sectional study
Source: BMC Public Health. 2024 Aug 29;24:2347. doi: 10.1186/s12889-024-19783-1 (PMC11360311; doi:10.1186/s12889-024-19783-1)
Supplement: Supplementary file 4 — Supplementary Material 4: Additional File 4. The adjusted association between workplace stressors and mental health during the first wave of the pandemic [file 12889_2024_19783_MOESM4_ESM.docx]

**Additional File 4.** The adjusted association between workplace stressors and mental health during the first wave of the pandemic

|  | Anxiety  Adjusted OR (95% CI) | Depression  Adjusted OR  (95% CI) | Disengagement  Adjusted OR (95% CI) | Exhaustion  Adjusted OR (95% CI) | Burnout  Adjusted OR (95% CI) |
| --- | --- | --- | --- | --- | --- |
| Felt overwhelmed by workload or family/work balance | **2.85 (1.53-5.31)** | **3.36 (1.66-6.79)** | **2.45 (1.27-4.72)** | **3.75 (1.93-7.27)** | **2.76 (1.51-5.05)** |
| Felt disconnected from family and friends because of workload | **3.17 (2.06-4.87)** | **2.96 (1.86-4.69)** | **1.79 (1.08-2.98)** | **5.48 (3.23-9.31)** | **3.01 (1.92-4.71)** |
| Felt inadequately compensated for work | **2.15 (1.48-3.11)** | **1.75 (1.20-2.57)** | **2.28 (1.42-3.66)** | **2.79 (1.66-4.67)** | **2.68 (1.75-4.09)** |
| Felt unappreciated at work | **2.72 (1.87-3.95)** | **2.01 (1.36-2.95)** | **4.07 (2.54-6.53)** | **3.15 (1.89-5.25)** | **3.65 (2.39-5.57)** |
| Experienced stigma or discrimination because of work | **2.04 (1.47-2.83)** | **1.64 (1.17-2.28)** | 1.31 (0.84-2.07) | 1.58 (0.95-2.62) | 1.31 (0.88-1.96) |
| Received job-related threats because of work | **1.92 (1.36-2.71)** | **1.50 (1.07-2.11)** | **1.77 (1.06-2.95)** | **2.32 (1.28-4.20)** | **1.96 (1.24-3.10)** |
| Felt bullied, threatened, or harassed because of work | **2.03 (1.46-2.81)** | **1.87 (1.34-2.60)** | **1.77 (1.12-2.78)** | **2.08 (1.25-3.46)** | **1.75 (1.17-2.63)** |
| Interacted often with the public | 1.10 (0.73-1.67) | 1.18 (0.76-1.82) | 0.80 (0.44-1.42) | 1.15 (0.62-1.12) | 0.94 (0.57-1.57) |
| Worried about workplace exposure to COVID-19 | 1.32 (0.95-1.83) | 1.36 (0.97-1.90) | **1.71 (1.08-2.72)** | **2.22 (1.32-3.75)** | **2.00 (1.32-3.02)** |

1. Adjusted for age, gender, ethnicity, education

Note: adjustment for potential confounders slightly changed estimates, only adjusted results are presented given the minimal difference in estimates. Bolded text indicates statistically significant findings.
